# Effects of cartilage-supporting nutritional supplementation on knee osteoarthritis symptoms and quality of life in a 12-week randomized double-blind placebo-controlled pilot study

**DOI:** 10.1038/s41598-025-11723-2

**Published:** 2025-07-15

**Authors:** Johannes-Paul Fladerer-Grollitsch, Thomas Klein, Albert Kompek, Nicole Steiner, Daniel Menzel, Christiane Schön

**Affiliations:** 1 Karl-Franzens-Universität Graz, Beethovenstraße 8, Graz, 8010 AT Austria; 2 Apomedica Pharmazeutische Produkte GmbH, Roseggerkai 3, Graz, 8010 AT Austria; 3 BioTeSys GmbH, Schelztorstraße 54-56, 73728 Essingen, GE Germany

**Keywords:** Osteoarthritis, Dr. Böhm^®^ Gelenks complex, Cartilage degradation, Nutritional intervention, Health care, Medical research

## Abstract

Osteoarthritis (OA) is a prevalent degenerative joint disease, often affecting the elderly, characterized by cartilage degradation and bone remodelling, leading to pain, stiffness, and impaired movement. This 12-week, randomized, double-blind, placebo-controlled pilot study investigated the efficacy of a formulation containing collagen type II, glucosamine hydrochloride and chondroitin sulfate, in alleviating knee OA symptoms in subjects with mild to moderate knee pain. Fifty-four participants were enrolled, with 52 completing the study. Participants were assessed using the Knee injury and Osteoarthritis Outcome Score (KOOS), performance-based physical function tests and global assessment tests. Results indicated significant improvements in the verum group compared to placebo. KOOS subscales showed improvements in symptoms (8.16%, *p* < 0.05), sport/recreation (25.25%, *p* < 0.01), and quality of life (27.66%, *p* < 0.001). Stiffness improved by 14.68% (*p* < 0.05) in the verum group. Both groups exhibited significant pain reduction (KOOS pain, *p* < 0.001 and *p* < 0.01 for verum and placebo, respectively). Performance-based measures, such as the Chair Stand test, improved significantly in both groups, though other metrics showed no significant differences. The verum group exhibited a linear improvement over 12 weeks, contrasting with the placebo group’s initial response and subsequent plateau. SF-36 Physical Component scores improved in both groups (*p* < 0.05), but mental health scores remained unchanged. Global assessment favoured the verum group, with more participants reporting OA improvement and recommending the product (*p* < 0.01). Biomarkers hsCRP and COMP showed no significant changes, likely due to sample variability. While the study did not achieve its primary endpoint, the nutritional formulation demonstrated an excellent safety profile and encouraging improvements in patient-reported symptoms and quality of life, warranting larger confirmatory trials.

**Registration number:** DRKS00029563 05/10/2022

**Supplementary Information:**

The online version contains supplementary material available at 10.1038/s41598-025-11723-2.

## Introduction

Osteoarthritis (OA) is a degenerative disease of the movable joints affecting the elderly population worldwide. It is characterized by localized loss of cartilage, remodelling of adjacent bone, and bony overgrowth because of a maladaptive cartilage repair process. Its presentation is highly variable between individuals, with some common features, such as joint pain, impaired movement, tenderness, crepitus, occasional effusion, and local inflammation^1,2^.

Besides hand and hip, the knees are frequently affected by OA whereas the prevalence is higher in women. Knee pain is a common complaint and prevalence increases with age^3^. Osteoarthritic knees show degeneration of the cartilage and pathological changes of the bone. Knee OA typically develops gradually over a period of years and is also termed degenerative osteoarthritis. Classical symptoms are pain, stiffness, limited range of motion and localized swelling. As knee osteoarthritis progresses, symptoms generally become more severe. The diagnosis of OA can usually be made on the basis of the initial history and examination. According to the American College of Rheumatology (ACR) radiographic and clinical knee OA diagnosis criteria include pain plus, at least, three of the following aspects: age over 50, morning stiffness (< 30 min), crepitus and presence of osteophytes. X-rays are typically used to confirm the diagnosis of osteoarthritis and can reveal osteophytes at the joint margins, joint space narrowing and subchondral bone sclerosis. The most common system to grade radiographic severity is the Kellgren and Lawrence grading system^2^.

There is no known cure for osteoarthritis, but treatments can help to reduce pain and maintain joint movement. Current recommendations for the management of OA include non-pharmacological interventions such as weight loss or exercise and pharmacological treatments with analgesics and anti-inflammatory drugs.

It is now increasingly recognized that, beyond meeting basic nutritional needs, nutrition may play a beneficial role in some diseases. Because the mechanisms of cartilage degradation in OA are multifactorial and some nutritional compounds usually contain multiple active compounds that target multiple pathways, nutrition could provide beneficial effects in the management of OA. Nutritional interventions are positioned to provide long-term rather than short-term effects. Although not a traditional inflammatory disease, symptoms of local inflammation and synovitis are present in many patients of OA. On this background, antioxidative and immune-modulating ingredients are recognized to be beneficial for OA. Several dietary supplements like glucosamine, chondroitin and collagen hydrolysate^4,5^ have demonstrated benefits compared to placebo and active controls. Even so the effectiveness of these compounds is under discussion as they are cartilage structure compounds providing reported synergistic effects^6–9^. Additionally, a recent meta-analysis describes small-to-moderate effects of collagen derivatives on pain alleviation (standardized mean difference [SMD] − 0.35, 95% confidence interval [CI] − 0.48 to − 0.22, moderate certainty) and function improvement (SMD − 0.31, 95%CI − 0.41 to − 0.22, high certainty) compared with the control in OA patients^10^.

In the tested formulation, the cartilage structure compounds are combined with vitamins and minerals to support the joint health synergistically. The aim of the pilot study was a proof of concept of the combination of substances to investigate the impact of the formulation on symptoms of OA patients with slight to moderate knee pain. To support symptom relief can greatly improve the quality of life and the mobility of the subject’s knees.

### Subject characteristics and ethics

On average, subjects in the Verum group were 62.7 years old (95% CI: 60.3–65.1) and in the Placebo group 59.4 years old (95% CI: 55.3–63.6). There were no differences in age between groups (*p* = 0.1417). Overall, 28 women (53.8%) and 24 men (46.2%) participated in the study. This was similarly distributed in both study groups. Participants in verum group had on average a slightly but significantly higher BMI of 26.40 kg/m² (95% CI: 25.23–27.58) in comparison to participants in the placebo group with BMI of 24.26 kg/m² (95% CI: 22.70–25.82). Most subjects (52.9%) in the verum group had OA grade III in accordance with Kellgren classification. Grade II was most present in the placebo group (44.4%). Overall, participants of the verum group were slightly older, the proportion of overweight subjects was higher (64.7% vs. 50%) and had a higher radiographic severity (KL-grade 3) (52.9% vs. 27.8%) than in the placebo group (Table 1). Nevertheless, no significant differences between verum and placebo group concerning the Kellgren scores could be observed (chi-square test: 4.045, df = 2, *p* ≈ 0.133).

**Table 1:** Subjects characertistics.

	Placebo	Verum
Gender (F/M)	10/8	18/16
Age	59.4 (± 8.4)	62.7 (± 7.0)
BMI	24.26 (± 3.14)	26.40 (± 3.37)
Kellgren score grade I	5	8
Kellgren score grade II	8	7
Kellgren score grade III	5	18

#### Ethical approval **and study design**.

This study was conducted in accordance with the guidelines for Good Clinical Practice (GCP) set forth by the International Council for Harmonisation of Technical Requirements for Pharmaceuticals for Human Use (ICH), and in accordance with the Declaration of Helsinki regarding the treatment of human subjects in a study. The study was registered in the German Clinical Trials Register (DRKS00029563).

#### Ethical approval

The ethical approval was obtained from the Institutional Review Board (IRB) of Landesärztekammer Baden-Württemberg on 18.05.2022.

### Conduct of the study

The study was performed as randomized, placebo-controlled, double-blind, pilot study over a period of 12 weeks to assess parameters of OA symptoms. Prior to any measurements and screening for eligibility, all subjects were informed in detail about the study procedure and signed informed consent. Medical history and chronic medication has been reported (STable 1 and STable 2).

In case all inclusion and none of the exclusion criteria were met, subjects were enrolled (STable 3). When subjects fulfilled all inclusion and none of the exclusion criteria, being eligible for the study they were allocated randomly (with allocation scheme of 2:1) to one of the two study groups (verum and placebo, respectively) according to the randomization list. The randomization scheme was created by using the software DatInf Randlist Version1.5 (DatInf GmbH Tübingen)^11^.

Study products (Dr. Böhm Gelenks complex as verum or cellulose tablets as placebo) were taken over a period of 12 weeks twice a day. Dr. Böhm Gelenks complex is a multinutrient food supplement with Glucosaminhydrochlorid, Chondroitinsulfate, Collagen complex with Type II collagen, mucopolysaccharids, hyaluronic acid, methylsulfonylmethan (MSM), selenium, manganese, vitamin C, vitamin E, vitamin D (Table 2). Verum and placebo had the same shape, color, smell and taste.

**Table 2:** Description of the investigational product dr. Böhm Gelenks complex.

Glucosaminhydrochlorid	350 mg	MSM	25 mg
Chondroitinsulfate	200 mg	Selenium	50 µg
Collagen-Complex natural from	100 mg	Mangan	1,0 mg
Collagen Type II	60 mg	Vitamin C	80 mg
Mucopolysaccarides	30 mg	Vitamin E	10 mg
Hyaluronic acid	10 mg	Vitamin D3	5,0 µg

Osteoarthritis symptoms were assessed with KOOS questionnaire at baseline, after 6 and 12 weeks of intervention. To assess physical function and judge symptoms under load, participants were asked to perform a recommended core set of performance-based tests at beginning and end of intervention. Time to complete the tests/or number of repetitions and pain under movement were assessed.

**Fig. 1:**
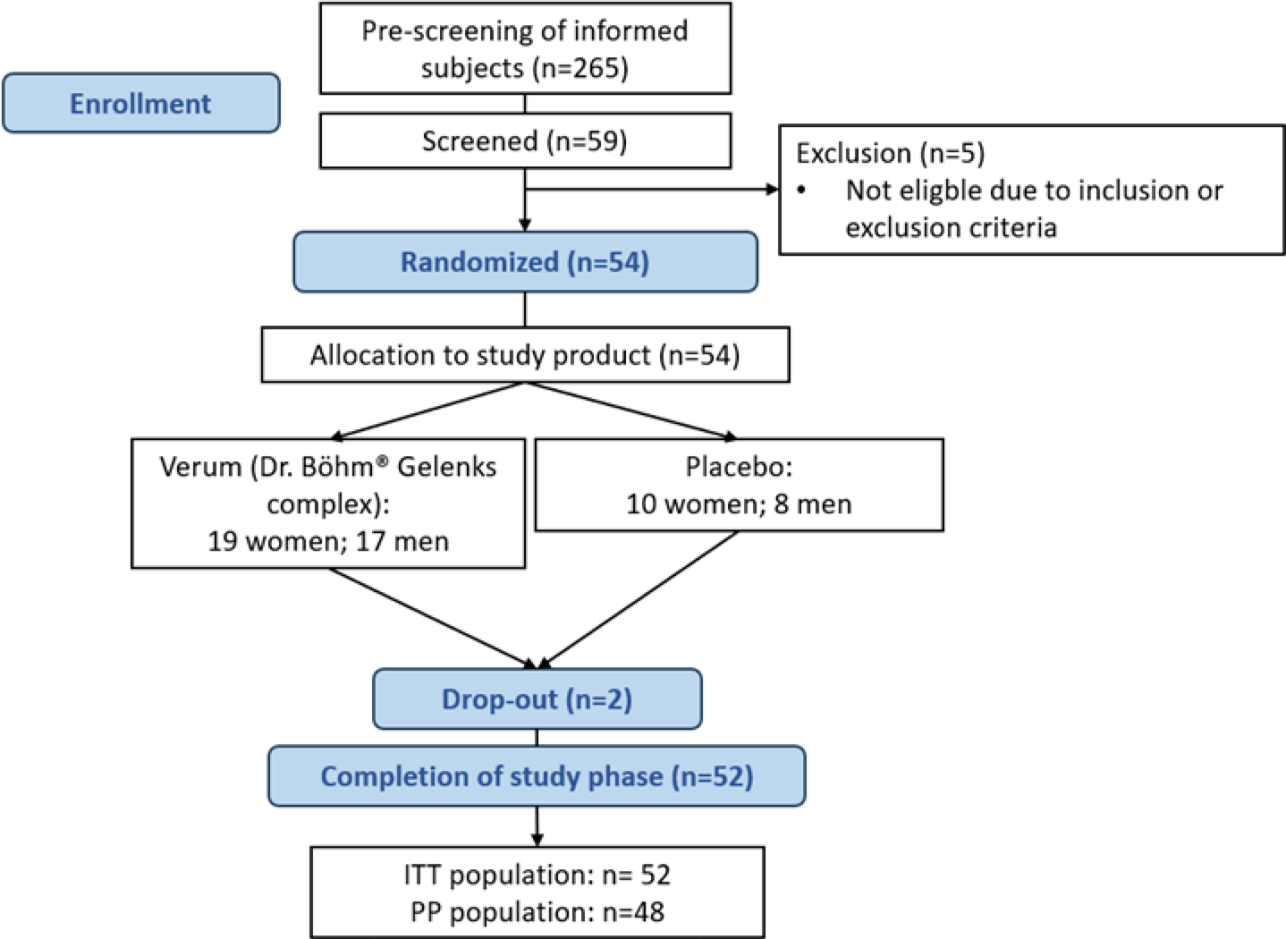
Disposition of subjects.

In the current study, 265 subjects received the study information and were pre-screened via phone, wherefrom 59 subjects were screened for eligibility. Thereof, 54 subjects were enrolled in the study, wherefrom 52 subjects completed the study in its entirety (Scr, visit 1-visit 3). The medical history and chronic medication is given in ATable 2 and ATable 3. 2 subjects dropped out during study conduct (see Fig. 1).

All clinical procedures were conducted by trained study personnel under the oversight of the study physician. OA diagnosis and Kellgren–Lawrence grading were confirmed at screening by a board-certified orthopaedist based on radiographic or MRI images. At each on-site visit (V2 and V3), participants first completed the KOOS questionnaire and a global assessment under the guidance of a trained study nurse, then immediately performed the 30-second chair stand, the 40 m fast-paced walk, and the stair climb tests in that order, with approximately 2 minutes’ rest between tests as per the study SOP. KOOS was always administered prior to any physical testing to avoid exercise-induced pain bias.

### KOOS questionnaire

KOOS is an extension of the Western Ontario and McMaster Universities Osteoarthritis Index (WOMAC) and is designed to survey more active people with knee injury and/or knee osteoarthritis^12^. The subscores pain, symptoms, stiffness, activity of daily life, sport and recreation, as well as quality of life were evaluated using a 5-point Likert scale from 0 (no problems) to 4 (extreme problems). The sum score was calculated as the sum of the items included. Scores were transformed to a 0–100 scale, with 0 (extreme knee problems) to 100 (no knee problems). In addition, in orientation to the WOMAC questionnaire, WOMAC subscores pain and stiffness were calculated from the KOOS questionnaire.

### Performance-based measures of physical function

In accordance with recommendations of OARSI^13^, the following performance-based tests were performed to assess physical function in individuals diagnosed with knee OA: 30-second chair stand test (maximum number of chair stand repetitions possible in a 30 s period are counted), 40 m fast paced walk test (fast-paced walking test over 4 × 10 m) and the stair climb test (SCT) (time it takes to ascend and descend a flight of stairs with 9 steps). Additionally, at rest before all performance-based tests and immediately after the SCT test, the level of pain in the target knee after was assessed using a Numeric rating scale (NRS: 0 (no pain) to 10 (extreme pain)). If both knees were affected by OA, the knee with more complaints was defined as the target knee. Time taken to complete the tests was recorded to the nearest 100th of a second with a digital stopwatch. A higher score indicated lower physical function.

### SF-36

The SF-36 questionnaire is a self-administered questionnaire containing 36 items which takes about 5 min to complete. It measures health on eight multi-item dimensions [Physical Functioning (PF), Role-Physical (RP), Bodily Pain (BP), General Health (GH), Vitality (VT), Social Functioning (SF), Role-Emotional (RE) and Mental Health (MH)], covering functional status and wellbeing, and overall evaluation of health. The scales were combined in a Physical Component Summary (PCS) and the Mental Component Summary (MCS).

### Global assessment

At baseline, V1, V2 and V3 patients had to judge the OA in the target knee within the last 48 h and the pain in the target knee within the last 4 weeks. At the end of the study, subjects were asked to rate the efficacy of the study product; to judge the OA in the target knee within the last 48 h compared to the beginning of the product intake; whether they would recommend the product to acquaintances/friends/family or whether they would continue to take the product.

### Obtaining of biological samples

At screening and visit 3, blood sample were taken to determine safety parameters and confirmation of the subjects’ state of health. Blood sampling was performed after at least 10 h overnight fast. To control for confounding factors, a standardized a standardized dinner was taken the days before. In addition, subjects are asked to keep their nutrition and exercise habits over the whole study period.

Blood routine parameters, such as liver enzymes (GPT, GOT, γ-GT, AP), fat status (total cholesterol, LDL- and HDL- cholesterol, triglycerides), creatinine, uric acid as parameters of kidney function, and differential haemogram were assessed. In addition, the biomarkers hsCRP and COMP (cartilage oligometric protein). All blood parameters were measured in an accredited laboratory. For vital signs and routine hematology and biochemistry, we observed a slight increase in mean eosinophil counts from baseline in the verum arm (+ 0.07 × 10^9/L versus + 0.02 × 10^9/L in placebo) and a modest rise in triglyceride levels (+ 0.15 mmol/L versus + 0.05 mmol/L). All other vital-sign and laboratory parameters remained within normal ranges. Causality for these laboratory findings was adjudicated by the study investigator using WHO criteria and deemed ‘unlikely’ related to the intervention.

### Safety, adverse events and concomitant medication

During the study intervention, subjects documented any adverse events (AE; unfavourable and unintended sign including an abnormal laboratory finding, symptom or disease) and concomitant medication in a subject’s diary. At each visit, changes in physical conditions, concomitant therapy, severity and outcome of AE were asked for, evaluated and recorded by investigator. All AEs were inquired and documented in accordance to ICH/GCP Guidelines in the electronical case report form (eCRF).

### Randomization and blinding

All subjects received a screening number at screening (S001, S002,…). When subjects fulfilled all inclusion and none of the exclusion criteria, being eligible for the study they were allocated randomly (with allocation scheme of 2:1) to one of the two study groups (verum and placebo, respectively) according to the randomization list by means of consecutive counting following the schedule of their inclusion visit. Subjects received subject numbers (P101, P102…). Assignment of subject to screening number/subject number was documented in subject identification list. The subject/screening numbers further identified the subjects and their treatments, documents, etc. Only the investigator and study coordinator were allowed to have access to this list.

Temporary interruption of IMP is instituted for reversible safety triggers (e.g., Grade ≥ 3 TEAEs or clinically significant lab abnormalities) and resumed once events return to ≤ Grade 1. Permanent withdrawal from the study occurs in cases of SAEs, persistent non-compliance, or participant request for discontinuation.

The randomization scheme was created by using the software DatInf Randlist Version1.5 (DatInf GmbH Tübingen). According to the randomisation list, the study products were labelled with the randomization number (subject number) and time point of hand out. The randomization list was kept at Sponsor site.

Only the investigator and study coordinator were allowed to have access to this list.

### Statistics

An intention-to-treat (ITT) analysis was performed. Delta changes between end of intervention and baseline and considering baseline as covariate was evaluated using ANCOVA statistics. To confirm results, the Student t-test was applied. If data were not normally distributed, the Mann Whitney test was applied. Baseline sclerotisation characteristics and change-from-baseline (CfB) descriptive statistics (mean ± SD) were calculated for both placebo and verum groups. Baseline group comparisons were performed using independent t-tests, while between-group differences in CfB were evaluated by ANCOVA with baseline values as covariates. ANCOVA and two-tailed t-test analysis were performed in SPSS 22 and a significance level of 0.05 was used. Within figures results are depicted as box plots or as bar charts if data represent descriptive information. Significance levels with *p* < 0.001 are indicated with ***, *p* < 0.01 with ** and *p* < 0.05 with *. For Global assessment questionnaire Chi square analysis and Fischer´s exact tests were performed. All statistics compared the efficacy after 12 weeks of treatment with baseline characteristics. To test for linearity of efficacy over time, R^2^ values have been calculated via Pearson´s regression model including baseline values and the effects after 6 and 12 weeks of treatment. Results are plotted to compare placebo and verum group.

## Results

### KOOS and WOMAC scores

**Fig. 2:**
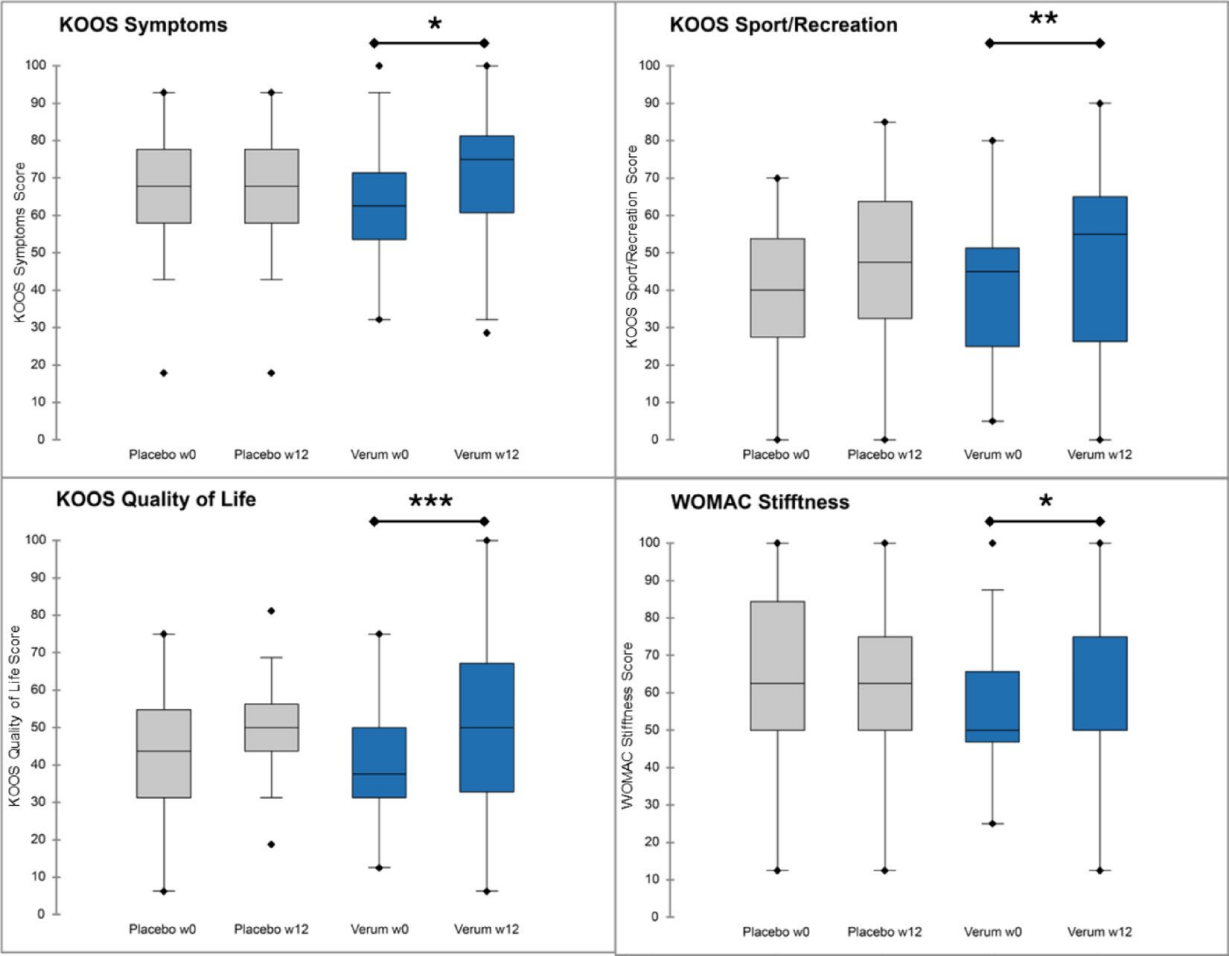
Comparison of placebo and verum group by KOOS questionnaire at baseline (w0) and after 12 weeks of intervention.

After 12 weeks of intervention, KOOS symptoms were significantly improved by 8.16% (*p* < 0.05), KOOS Sport/Recreation score by 25.25% (*p* < 0.01), KOOS Quality of Life by 27.66% (*p* < 0.001) and WOMAC Stiffness by 14.68% (*p* < 0.05) in the verum group. In contrast to that, the placebo group did not provide any significant improvements (Fig. 2).

The KOOS Pain score was significantly improved in the verum group by 19,1% (*p* < 0.001) as well as in the placebo group by 16,7% (*p* < 0.01). The same could be found for KOOS Activities of daily Life (verum: *p* < 0.001; placebo: *p* < 0.01) and WOMAC Pain (improvement verum 22,4% and placebo 18,9%: *p* < 0.001). However, delta changes after the 12-week intervention between groups did not show significant differences (ANCOVA: *p* = 0.6649), and therefore the primary endpoint was not met.

### Performance-based measures of physical function

After 12 weeks of intervention, both the verum and placebo groups showed significant improvements in chair stand repetitions (verum: *p* < 0.001; placebo: *p* < 0.05), with no significant between-group difference. No significant changes were observed in 40 m walk test speed or in pain scores before or during the performance test. These parallel gains indicate that Dr. Böhm^®^ Gelenks complex is safe and well tolerated but did not outperform placebo on these functional measures, highlighting the need for larger trials to determine any true treatment effect.

### SF-36

The SF-36 PCS was significantly improved (p $<$ 0.05) in both groups. In contrast to that, the SF-36 MCS did not show any significant changes.

### Global assessment

**Fig. 3:**
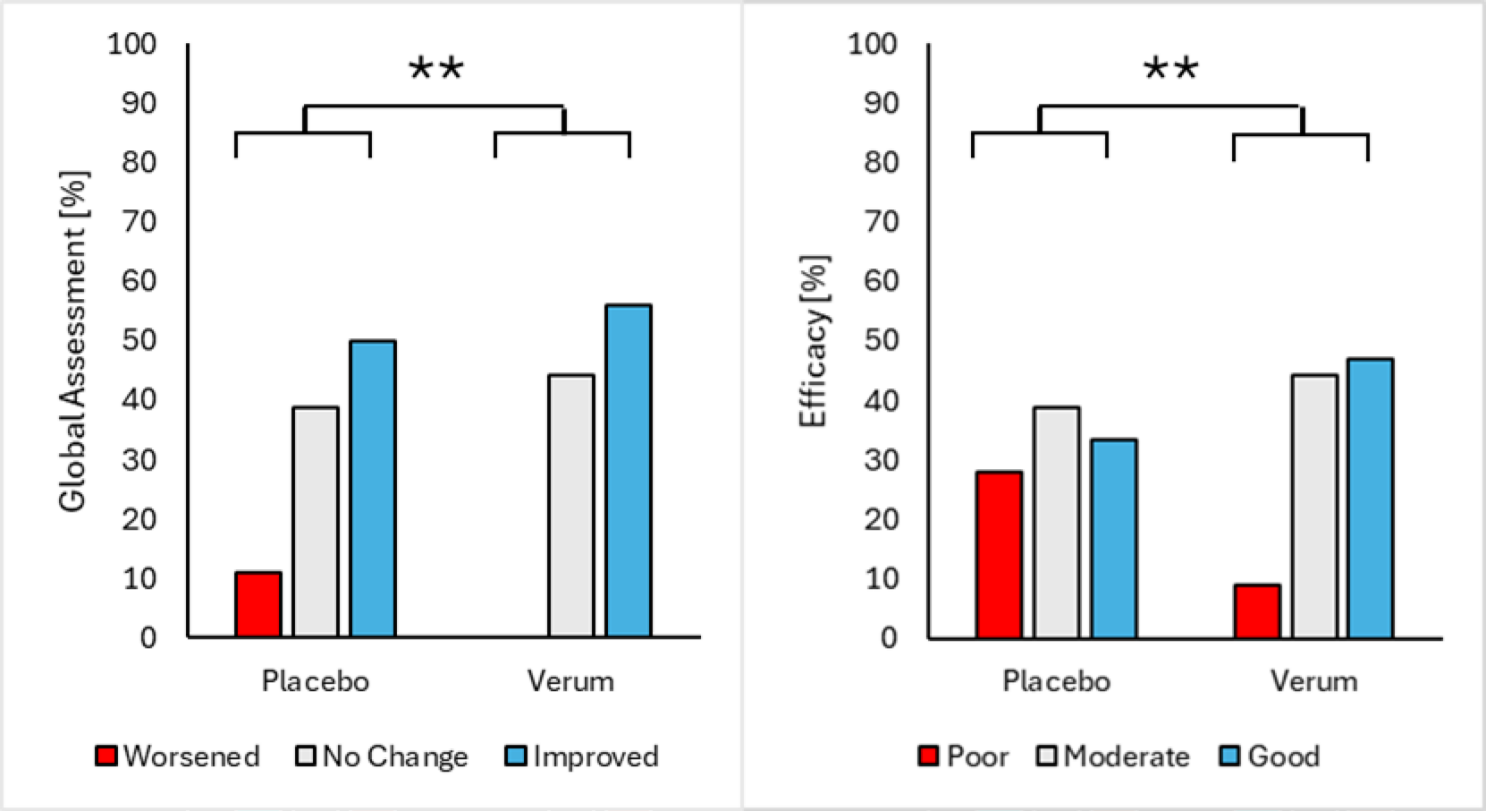
Comparison of verum and placebo concerning their Global Assessment and Efficacy of study product.

The observed distribution of categorical responses of the Global Assessment questionnaire was significantly (Fischer exact test: *p* < 0.01) increased in the verum group compared with placebo group with more people judging their osteoarthritis as improved. Similar results could be observed for the rating of product efficacy with a significant better outcome for the verum group (Fischer exact test: *p* < 0.01). (Fig. 3)

**Fig. 4:**
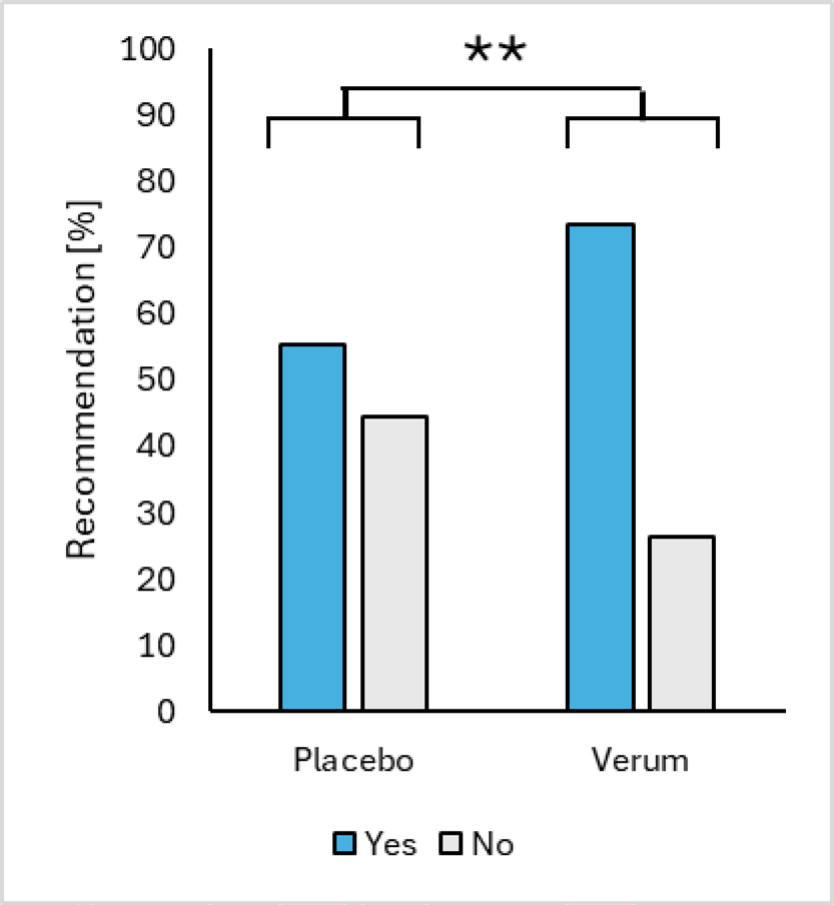
Recommendations of verum and placebo group after the 12 weeks of treatment.

Going along with these results, in the verum group significantly more subjects would recommend further intake of the study product to relatives/friends (Chi square test: *p* < 0.01) (Fig. 4).

### HsCRP and COMP biomarkers

No significant effects of verum or placebo could be observed on hsCRP and COMP biomarkers. This may be according to the limited sample size and the large variability of these parameters. (Table 3)

**Table 3:** HsCRP and COMP biomarkers.

	Placebo		Verum	
	Baseline	12 weeks	Baseline	12 weeks
hsCRP	1.332 (± 1.079)	1.149(± 1.323)	1.897 (± 2.683)	1.603 (± 1.480)
COMP	10.806 (± 2.606)	10.264 (± 2.389)	11.094 (± 2.636)	11.304 (± 3.048)

### Safety

Subjects rated overall tolerability as very good. During the study no serious adverse event occurred. In the placebo group (*n* = 18) 57 adverse events have been reported, while in the verum group (*n* = 36) 41 adverse events occurred. Adverse events (AEs) were assessed in all subjects who received ≥ 1 dose (Placebo *N* = 18; Verum *N* = 36). Causality was adjudicated by the study investigator using WHO terminology (“unlikely” 5–25% probability; “possible” > 25–50% probability). Serious adverse events (SAEs) are not graded for causality; no SAEs occurred in this study.

Overall, 98 treatment-emergent AEs (TEAEs) were reported by 29 subjects: 57 events in 11 placebo subjects (61.1%) versus 41 events in 18 verum subjects (50.0%). Related AEs (WHO “unlikely”: 10 events; “possible”: 2 events) occurred in 2 placebo subjects (11.1%) and none in the verum arm.

Only System Organ Classes (SOCs) with event frequencies in ≥ 5% of subjects in at least one arm are shown below. Preferred Terms (PTs) occurring in ≥ 5% of subjects and grade-wise breakdown require individual-listing review for exact subject-level counts (Table 4 and STable 4).

**Table 4:** Summary of treatment-emergent adverse events (TEAEs) by system organ class (SOC) during the 12-week intervention (V1–V3) in the safety population (subjects receiving ≥ 1 dose). Data are number of events, (number of subjects with TEAEs) and [percentage] of subjects per arm.

SOC/Preferred Term	Placebo *n* = 18	Verum *n* = 36	Total *n* = 54
Nervous system disorders	11 (4) [22.2%]	17 (9) [25%]	28 (13) [24.1%]
Gastrointestinal disorders	15 (4) [22.2%]	6 (5) [13.9%]	21 (9) [16.7%]
Musculoskeletal & connective tissue disorders	13 (5) [27.8%]	5 (5) [13.9%]	18 (10) [18.5%]
Respiratory, thoracic & mediastinal disorders	9 (6) [33.3%]	8 (8) [22.2%]	17 (14) [25.9%]
General disorders & administration-site conditions	7 (4) [22.2%]	1 (1) [2.8%]	8 (5) [9.3%]
Injury, poisoning & procedural complications	0	2 (1) [2.8%]	2 (1) [1.9%]
Renal & urinary disorders	1 (1) [5.6%]	0	1 (1) [1.9%]
Eye disorders	0	1 (1) [2.8%]	1 (1) [1.9%]
Ear & labyrinth disorders	0	1 (1) [2.8%]	1 (1) [1.9%]
Surgical & medical procedures	1 (1) [5.6%]	0	1 (1) [1.9%]
Total TEAEs (events)	**57**	**41**	**98**
Subjects with ≥ 1 TEAE	11 [61.1%]	18 [50.0%]	29 [53.7%]
Subjects with related TEAE	2 [11.1%]	0	2 [3.7%]
SAEs	0	0	0

## Discussion

The results of this pilot study suggest that the combination of substances in Dr. Böhm^®^ Gelenks complex has a positive impact on alleviating the symptoms associated with knee osteoarthritis (OA), particularly in individuals experiencing slight to moderate knee pain. Other preparations with collagen-containing components, such as the hydrolyzed collagen evaluated by Carrillo-Norte et al., have shown comparable benefits in knee OA: in their six-month, randomized, double-blind, placebo-controlled trial, daily oral hydrolyzed collagen significantly improved WOMAC pain and function scores versus placebo^14^. These parallel findings reinforce the potential of collagen-based nutritional supplements to alleviate symptoms and enhance function in patients with knee OA, and support the need for larger, confirmatory trials. The KOOS and the WOMAC are established tools for assessing the condition^15^ and symptoms of individuals with knee OA^12^. In this study, significant improvements were observed in the KOOS symptoms, Sport/Recreation, and Quality of Life subscales in the verum group compared to the placebo group. Specifically, the KOOS symptoms score improved by 8.16% (*p* < 0.05), the Sport/Recreation score by 25.25% (*p* < 0.01), and the Quality of Life score by 27.66% (*p* < 0.001). These improvements indicate that the verum intervention positively impacts both the functional and quality of life aspects of knee OA.

**Fig. 5:**
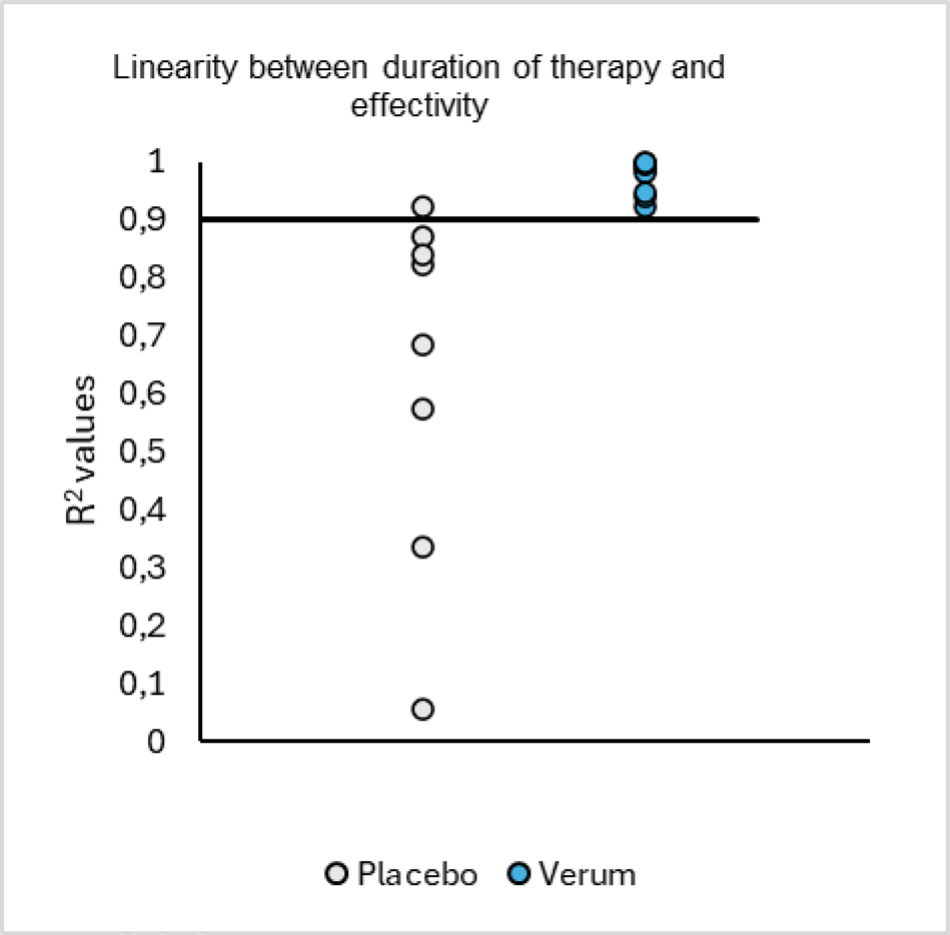
R² values indicating goodness-of-fit for linear models across four assessment time points for each outcome (KOOS Pain, KOOS Symptoms, KOOS ADL, KOOS Sport/Recreation, KOOS QoL, WOMAC Pain, WOMAC Stiffness, and Chair Stand Repetition); lower R² denotes more non-linear trajectory.

Furthermore, the stiffness score in orientation to the WOMAC subscale showed a significant improvement of 14.68% (*p* < 0.05) in the verum group. This improvement in stiffness can contribute to better joint mobility and reduced discomfort during daily activities. The KOOS Pain score was significantly improved in both the verum (*p* < 0.001) and placebo (*p* < 0.01) groups, although the magnitude of improvement was greater in the verum group. Similarly, the KOOS Activities of Daily Living and Pain scores improved significantly in both groups (*p* < 0.001). Furthermore, performance-based measures of physical function, such as the Chair Stand test, showed significant improvements in both the verum (*p* < 0.001) and placebo (*p* < 0.05) groups. However, other performance metrics like the speed of 40 m fast paced walk test, duration of SCT, pain prior and during performance-based measures^13^ did not show significant differences between groups. Interestingly, with the exception of the chair stand repetition test, in all other cases showing significant improvements in the verum and the placebo group, the placebo led to an increased effect during the first 6 weeks of treatment followed by a stationary or decreased effectivity afterwards. This goes along with previous findings pointing out that in pain treatment the placebo effect can last over many days but is decreased over longer time^16^. In contrast, the verum group provided a linear increase in effectivity over 12 weeks in all cases. These data are highlighted in Fig. 5, demonstrating the different time course of verum and placebo by comparing the R^2^ values of linear regressions. R² values reflect the degree to which each outcome’s four-visit trajectory approximates linearity, providing a comparative measure of temporal consistency across endpoints.

These findings suggest a stable and linear long-time effectivity of the verum compared to the placebo.

The SF-36 Physical Component Summary was significantly improved in both the verum (*p* < 0.05) and placebo (*p* < 0.05) group, indicating that both interventions had a positive effect on the physical aspects of health-related quality of life. However, no significant changes were observed in the SF-36 Mental Component Summary, which might be attributed to the primary focus of OA symptoms on physical health rather than mental health. Despite the lack of change in mental health scores, the overall physical health improvements in the verum group underscore the potential of Dr. Böhm^®^ Gelenks complex in enhancing the physical well-being of individuals with knee OA.

The Global Assessment results revealed a significant difference between the verum and placebo group, with the verum group showing a greater proportion of participants reporting improvement in their OA condition (Fischer exact test: *p* < 0.01). Additionally, the efficacy of the product was rated significantly higher in the verum group (Fischer exact test: *p* < 0.01), and a greater number of participants in the verum group recommended the product (Chi square test: *P* < 0.01). These subjective assessments highlight the perceived benefits of the verum intervention in managing OA symptoms and enhancing overall joint health.

The biomarkers hsCRP and COMP did not show significant changes in either group. COMP is a high molecular weight, multi-submit protein originally isolated from cartilage. The protein is abundant in cartilage but is also found in tendon and other tissues^17,18^. In OA patients, serum COMP is increased with active disease progression^19^. In patients with knee OA, COMP levels correlate with the degree of synovial proliferation and osteophytosis but not with the femoral cartilage thickness^20^. Moreover, COMP is a marker of joint metabolism and damage in both disease and sport^21^. This high variability and limited sample size could have contributed to these non-significant findings^22^. However, the absence of significant changes in these biomarkers does not necessarily negate the clinical benefits observed through subjective and functional assessments. The overall tolerability of the intervention was rated as very good, and no serious adverse events were reported during the study, indicating that Dr. Böhm^®^ Gelenks complex is safe for use in this population.

The verum group consistently demonstrated superior outcomes across multiple domains, including significant improvements in KOOS and derived calculated WOMAC subscores, performance-based measures, and global assessments of efficacy and recommendation. These results suggest that the combination of cartilage structure compounds, vitamins, and minerals in Dr. Böhm^®^ Gelenks complex provides a more comprehensive and effective approach to managing knee OA symptoms compared to placebo, offering a promising treatment to manage OA. To better account for time-dependent improvements often observed in OA, future trials might incorporate a randomized placebo run-in followed by long-term withdrawal or an analgesic baseline analgesic usage assessment with down-tapering. However, as this was a pilot study, such design modifications were not feasible and will be considered in subsequent confirmatory trials. No pain diary was included in this pilot but could be implemented in confirmatory studies to enhance symptom tracking.

### **Limitations**

The current study was conducted as a pilot study and the sample size was rather small, which may have limited the significance of the results. For future studies, a higher samples size may be more appropriate. One major limitation of this study is the unequal sample sizes between the placebo group (*n* = 18) and the verum group (*n* = 36). This imbalance may affect the statistical power and reliability of the comparisons between the groups. Smaller sample sizes, such as that of the placebo group, are more susceptible to random variation, which could skew the results or reduce the ability to detect subtle differences. Future studies should aim to recruit balanced sample sizes to ensure more robust and generalizable results.

Only subjects with radiographic finding of Kellgren Score I – III were allowed to participate. An additional limitation is the baseline difference in KL grade severity. Although the difference was not significant, the imbalance in KL grade severity may still influence the results.

## Conclusions

In summary, although the primary endpoint was not met, exploratory analyses indicate a trend toward improvement in symptoms and quality of life with Dr. Böhm^®^ Gelenks complex in mild to moderate knee OA. The significant improvements observed in the verum group across various measures underscore the potential of nutritional interventions in OA management. Further studies with larger sample sizes and longer follow-up periods are warranted to confirm these findings and elucidate the underlying mechanisms of action. However, the unequal sample sizes between the groups (*n* = 36 for verum, *n* = 18 for placebo) may limit the robustness and generalizability of this conclusion. As per our randomized-placebo‐controlled design, the single‐blind dose‐escalation step was implemented to minimize placebo response. Nonetheless, the current results highlight the promise of a multimodal nutritional approach in providing sustained symptom relief and enhancing joint health in knee OA patients.

## Electronic supplementary material

Below is the link to the electronic supplementary material.

Supplementary Material 1

## Data Availability

Data are available on request (johannes.fladerer@uni-graz.at).
