# Supplementary material for: Effects of cartilage-supporting nutritional supplementation on knee osteoarthritis symptoms and quality of life in a 12-week randomized double-blind placebo-controlled pilot study
Source: Sci Rep. 2025 Jul 15;15:25625. doi: 10.1038/s41598-025-11723-2 (PMC12264193; doi:10.1038/s41598-025-11723-2)
Supplement: Supplementary file 1 — Supplementary Material 1 [file 41598_2025_11723_MOESM1_ESM.docx]

**Supplemental Material**

**STable 1: Medical history**

| **Medical History, Frequency [n]** | |  |  |  |
| --- | --- | --- | --- | --- |
| **Category** | **Description** | **Placebo (n=18)** | **Verum (n=36)** | **Total (n=54)** |
| **B** | Neoplasms benign, malignant and unspecified (incl cysts and polyps) |  | 1 | 1 |
| **C** | Blood and lymphatic system disorders |  | 3 | 3 |
| **D** | Immune system disorders | 5 | 13 | 18 |
| **E** | Endocrine disorders | 3 | 3 | 6 |
| **F** | Metabolism and nutrition disorders | 3 | 8 | 11 |
| **G** | Psychiatric disorders | 1 | 2 | 3 |
| **H** | Nervous system disorders |  | 2 | 2 |
| **I** | Eye disorders |  | 1 | 1 |
| **J** | Ear and labyrinth disorders |  | 2 | 2 |
| **L** | Vascular disorders | 4 | 12 | 16 |
| **M** | Respiratory, thoracic and mediastinal disorders | 3 | 2 | 5 |
| **O** | Hepatobiliary disorders |  | 1 | 1 |
| **P** | Skin and subcutaneous tissue disorders |  | 1 | 1 |
| **Q** | Musculoskeletal and connective tissue disorders | 2 |  | 2 |
| **R** | Renal and urinary disorders |  | 1 | 1 |
| **T** | Reproductive system and breast disorders |  | 1 | 1 |
| **Total** |  | **21** | **53** | **74** |
| **Reported by** | | **10 subjects** | **27 subjects** | **37 subjects** |

**STable 2: Chronical medication**

| **Chronic Medication, Frequency [n]** | |  |  |  |
| --- | --- | --- | --- | --- |
| **ATC code** | **Description** | **Placebo (n=18)** | **Verum (n=36)** | **Total (n=54)** |
| **A11** | Vitamins | 2 | 4 | 6 |
| **A12** | Mineral supplements |  | 1 | 1 |
| **A14** | Anabolic agents for systemic use |  | 1 | 1 |
| **B01** | Antithrombotic agents | 2 | 1 | 3 |
| **C03** | Diuretics |  | 1 | 1 |
| **C07** | Beta blocking agents |  | 2 | 2 |
| **C08** | Calcium channel blockers | 1 | 5 | 6 |
| **C09** | Agents acting on the renin-angiotensin system | 3 | 9 | 12 |
| **C10** | Lipid modifying agents | 3 | 6 | 9 |
| **G02** | Other gynecological agents | 1 |  | 1 |
| **G03** | Sex hormones and modulators of the genital system |  | 2 | 2 |
| **G04** | Urologicals |  | 2 | 2 |
| **H03** | Thyroid therapy | 3 | 7 | 10 |
| **N05** | Psycholeptics | 1 |  | 1 |
| **N06** | Psychoanaleptics |  | 2 | 2 |
| **R01** | Nasal preparations | 1 |  | 1 |
| **S01** | Ophthalmologicals |  | 1 | 1 |
| **V06** | General nutrients | 1 |  | 1 |
| **Total** |  | **18** | **44** | **62** |
| **Reported by** | | **10 subjects** | **21 subjects** | **31 subjects** |

**STable 3 Inclusion/Exclusion criteria**

| **Inclusion criteria** | **Exclusion criteria** |
| --- | --- |
| Subject is able and willing to sign the Informed Consent Form prior to screening evaluations | Inflammatory, infectious or metabolic joint disorder |
| Sex: female and male (minimum 25% of each gender) | Artificial joint replacement in knees or hip |
| Subjects with primary osteoarthritis who are not expected to require surgical treatment for at least three months after inclusion. | Surgery within the last 6 months in knees or hip |
| Known X-ray or MRI finding (Kellgren score of 1 to 3; X-ray or MRI not older than 36 months) | Intra-articular therapy within the last 3 months |
| Discomfort over a period of at least 3 months | Oral cartilage treatment within the last 3 months including also supplements e.g. glucosamine, chondroitin, hyaluronic acid or collagen. |
| KOOS pain score with maximum 75 (Maximum 100 (no pain problems)) at screening | Medical treatment of joint disease besides Paracetamol as analgesic medication for emergency treatment |
| Age: 40 – 75 years | Anticipating any planned changes in lifestyle for the duration of the study |
| BMI: 19 – 32 kg/m2 | Known allergy and hypersensitivity to ingredients of the study product (e.g. crustacea) |
| Subjects agree to maintain physical activity levels throughout the study | Blood donation within 1 month prior to study start or during study |
| Except for osteoarthritis, the subject is in good physical and mental health as established by medical history, physical examination, vital signs, results of biochemistry, hematology | Relevant history or presence of any severe medical disorder, potentially interfering with this study (e.g. mal absorption, chronic gastro-intestinal diseases (e.g. Morbus Crohn, Colitis Ulcerosa), heavy depression, significant cardiovascular disease or co-morbidities (e.g. myocardial infarction, stroke, congestive heart failure), diabetes, acute malignant disease within last 3 years except basal cell carcinoma of the skin, etc.) |
| Subject is able to communicate well with the Investigator, to understand and comply with the requirements of the study, and be judged suitable for the study in the opinion of the Investigator | Present or recent use of drugs and dietary supplements with anti-inflammatory properties that could interfere with the study 2 months before or during the study (e.g. Curcumin, omega-3 FA, chronic intake of NSAR, ibuprofen, glucocorticoids (except inhalative) etc.), antioxidative supplements (e.g. vitamin C, vitamin E) or further antioxidative supplements (e.g. OPC etc.). |
|  | Subjects with history of drug, alcohol or other substance abuse, or other factors limiting their ability to co-operate during the study. |
|  | Known pregnancy, breast feeding or intention to become pregnant during the study. A pregnancy test will be conducted during screening and visit 3. |
|  | Participation in another clinical study within the last 4 weeks and concurrent participation in another clinical study |
|  | Weight loss intervention or recent body weight change > 5% during last 3 months |
|  | Subjects considered inappropriate for the study by investigators, including subjects who are unable or unwilling to show compliance with the protocol. |

**STable 4: Adverse events**

| **Adverse events during intervention (V1 - V3), Frequency [n]** | |  |  |  |
| --- | --- | --- | --- | --- |
| **ATC code** | **Description** | **Placebo (n=18)** | **Verum (n=36)** | **Total (n=54)** |
| **H** | Nervous system disorders | 11 | 17 | 28 |
| **I** | Eye disorders |  | 2 | 1 |
| **J** | Ear and labyrinth disorders |  | 2 | 1 |
| **M** | Respiratory, thoracic and mediastinal disorders | 9 | 8 | 17 |
| **N** | Gastrointestinal disorders | 15 | 6 | 21 |
| **Q** | Musculoskeletal and connective tissue disorders | 13 | 5 | 18 |
| **R** | Renal and urinary disorders | 1 |  | 1 |
| **V** | General disorders and administration site conditions | 7 | 1 | 8 |
| **X** | Injury, poisoning and procedual complications |  | 2 | 2 |
| **Y** | Surgical and medical procedures | 1 |  | 1 |
| **Total** |  | **57** | **41** | **98** |
| **Reported by** | | **11 subjects** | **18 subjects** | **29 subjects** |
